# Stereotypes of Social Groups in Mainland China in Terms of Warmth and Competence: Evidence from a Large Undergraduate Sample

**DOI:** 10.3390/ijerph18073559

**Published:** 2021-03-30

**Authors:** Zouhui Ji, Yaping Yang, Xinfang Fan, Yuting Wang, Qiang Xu, Qing-Wei Chen

**Affiliations:** 1 Department of Psychology, Ningbo University, Ningbo 315211, China; 19210730093@fudan.edu.cn (Z.J.); 1911041210@nbu.edu.cn (X.F.); 186002485@nbu.edu.cn (Y.W.); xuqiang1@nbu.edu.cn (Q.X.); 2 Department of Psychology, Fudan University, Shanghai 200433, China; 3 Lab of Light and Physiopsychological Health, National Center for International Research on Green Optoelectronics, South China Normal University, Guangzhou 510006, China; 4 Guangdong Provincial Key Laboratory of Optical Information Materials and Technology & Institute of Electronic Paper Displays, South China Academy of Advanced Optoelectronics, South China Normal University, Guangzhou 510006, China; 5 School of Psychology, South China Normal University, Guangzhou 510631, China

**Keywords:** stereotype content model, warmth, competence, China

## Abstract

The Stereotype Content Model (SCM) has been validated in multiple countries and regions. However, previous validation studies in China have been limited by small sample size. The current research increased the sample size (n = 184 in the pilot study; n_1_ = 1315 and n_2_ = 268 in the formal study) to validate the SCM in mainland China in study 1. Supporting the SCM, 41 social groups were clustered into four quadrants based on warmth and competence dimensions. 35 of the 41 target groups (85.37%) receive ambivalent stereotype. Perceived warmth and competence were positively correlated (*r* = 0.585, *p* < 0.001). Status and competence were positively related (*r* = 0.81, *p* < 0.001), and competition and warmth were negatively related (*r* = −0.77, *p* < 0.001). In addition, 24 typical social groups were selected and a list of stereotype words for these groups was developed in study 2 (n_1_ = 48, n_2_ = 52). The implications of the emerging social groups and the applications of this stereotype word list are discussed.

## 1. Introduction

### 1.1. Stereotype Content Model

Evaluating oneself, other individuals, in-groups, and out-groups happens all the time in everyday life, and multiple theories have been proposed to explain this complicated process [1,2]. The Stereotype Content Model (SCM) is one of the most well-known and influential theories and was first proposed by Susan Fiske and her colleagues [3]. According to the SCM, when someone encounters a new group, they evaluate them based on two dimensions: warmth and competence. Warmth is the group’s perceived intent and how likely they are to render help or impose harm. Competence is their ability to carry out that objective. Depending on the warmth-competence evaluation, social groups can be clustered into four quadrants: High Warmth-High Competence (HW-HC), High Warmth-Low Competence (HW-LC), Low Warmth-High Competence (LW-HC), and Low Warmth-Low Competence (LW-LC). Most groups receive ambivalent stereotypes and fall into the LW-HC or HW-LC quadrant [4,5], which means the majority of social groups are rated as high in one dimension and low in the other dimension. The results of the categorization of social groups determine how others perceive and treat them.

The SCM has been tested and validated in multiple countries and regions [4,5,6,7,8], such as Germany [9], Norway [10], India [11], and Romania [12]. Recently, the SCM has been introduced to eight post-communist societies [13]: Russia [14], Belarus, Ukraine, Kosovo, Uzbekistan, Kazakhstan, Georgia, and Armenia, which has greatly extended the SCM’s application in underrepresented regions of the world. Although some variations were revealed, the universality of competence and warmth has been largely supported in previous studies [13,15,16,17,18]. Despite the lack of variance between different places, the SCM predictions have shown their robustness over time [6,19], as well as under different conditions of applied research methods [20] and regardless of the method of data collection: representative samples [21], convenience samples [3], and offline or online data collection [22]. Demographic differences within a country do not affect the results [21]. Besides group perceptions, the SCM could also be applied to understand interpersonal perceptions [23,24,25,26] and self-evaluation [27]. In addition to human applications, the SCM could be applied to non-human entities, such as animals [28,29], brands [30,31,32,33,34], and countries [35,36,37]. Due to the universality of warmth and competence, the SCM has been widely used as a theoretical framework in multiple fields, such as political science [38], clinical settings (such as [39,40], see [41] for a review), tourism (e.g., [42,43,44,45]) and the marketing field (for review, see [46]).

### 1.2. The Content of Stereotypes in China

Validation of the SCM in China has attracted much attention since it was proposed and the related studies are summarized in Table 1. Fiske and her colleagues first tested the SCM in Hong Kong in 2009 [6] and updated the results in mainland China recently [47]. Four clusters (HW-LC, LW-HC, LW-LC, and MW-MC) were found in their 2009 study [6], while social groups were merged into six clusters (HW-HC, LW-LC, LW-MC, MW-HC, HW-MC, and MW-LC) in their recent investigation [47]. These significant differences mirrored the within-culture variation recently found in the SCM in Romania [12], and may be due to the cultural difference between Hong Kong and mainland China [48]. Despite the explorations of Fiske’s team, much effort has been devoted by scholars from mainland China; their research was not published in English and thus attracted less attention. The majority of their studies replicated the classical four-cluster findings of the SCM (HW-LC, LW-HC, LW-LC, and HW-HC) [49,50,51,52], though some variations were evident in these findings. For example, Gao (2010) found that a two-dimensional framework did not fit his data well and that a new dimension (morality) should be introduced into the SCM framework to better explain the findings [49]. For government workers, Guan and Cheng (2011) grouped them into the LW-HC cluster [50], while Shi and Wang (2017) classified this group into the LW-LC cluster [52].

These discrepancies might be due to the following factors. First, the cultural difference between Hong Kong and mainland China may have an effect, as mentioned above. Second, the different sets of groups for comparison may be another relevant factor. Intergroup perceptions would be affected substantially by comparative context [53,54]; thus, the location of each social group on the SCM map would be determined by the social groups selected by the pretest, which were quite different among previous studies, although some groups overlapped (see Table 1).

### 1.3. The Current Research

Over the past half-century, unprecedented societal transformations have taken place in China. Such rapid societal change exerts a great influence on Chinese culture and psychology (see [55] for a review), such as narcissism [56], need for uniqueness [57], and self-esteem [58]. The change in cultural orientation [59,60,61,62] should be noted. More specifically, a decrease in collectivism and increase in individualism have been revealed among Chinese youths [59,60,61], which has made them become bicultural. These changes not only benefit multiple mental performances (such as opportunity recognition, evaluation, and exploitation) but also change social perception profoundly [63,64,65]. Considering the cultural variation found in the SCM in previous studies, the change in cultural orientation over recent years in China should be taken into account when investigating the SCM, and the SCM map might have changed over recent years. Moreover, some groups evaluated ten years ago do not exist or are not appropriate in China nowadays, such as “Teenagers born in the 80s” [49]. Some new social groups (e.g., urban management officers) have emerged and been reported in the media frequently during recent years, and more attention should be paid to them. Even for the same social group, the cultural stereotype may have been subject to significant change during recent years due to media reports. Given all the aforementioned factors, the stereotype content of social groups in mainland China needs to be updated and reevaluated.

Compared to competence-related information, warmth-related information has priority during social interaction, which is called the “primacy-of-warmth effect” [17,66]. However, the relationship between warmth and competence is much more complicated than that. The “primacy-of-warmth effect” suggests that warmth is likely to affect competence. However, recent evidence demonstrated that competence was as important as warmth when shaping impressions [67] and that competence-related information even positively influenced evaluation of warmth [68], which suggests that the correlation between warmth and competence may be bidirectional. The reported positive association between warmth and competence [4] confirmed this prediction. Conversely, compensation has been shown to occur between warmth and competence, especially when the context involved comparison (e.g., [69], see [70] for a review). Specifically, when comparing two targets, if the first target was rated high in one dimension (e.g., warmth), then the second target would be evaluated positively on the other dimension (e.g., competence). Previous Chinese studies reported no significant correlation between warmth and competence (r = 0.12 [6] and r = 0.18 [47]), which was not entirely consistent with findings from other countries [5]. With the development of the economy and the change in cultural values over the last decade in China, how the relationship between warmth and competence has changed over recent years needs to be elucidated.

According to the SCM, social structure (status and competition) predicts cultural stereotypes (warmth and competence) [3,21,71,72]. More specifically, perceived group status is positively associated with competence, while perceived intergroup competition is negatively correlated with warmth. The positive correlation between status and competence has been well supported in previous studies in China [3,21,71,72]. However, the negative association between competition and warmth only achieved partial support. Some studies confirmed this relationship [6,47,49,50], while others failed to reveal such an association [51,52]. Thus, the correlation between perceived competition and warmth needs to be determined.

To maintain consistency with the majority of previous studies, we recruited undergraduates in mainland China and collected data with paper-pencil questionnaires. The primary differences between the current investigation and previous ones lie in the sample size and the year in which the research was conducted. We increased the sample size in both the pilot study and formal study to increase the representativeness of social groups and the reliability and generalization of results. Additionally, the most recent study with an undergraduate sample in China was conducted more than a decade ago [50], while the current investigation was investigated in 2018, thus reflecting the latest state of the SCM map in China.

The main purpose of the present investigation was to validate the SCM and update the stereotype content in mainland China; this was explored in study 1. An additional aim was to develop a stereotype word list for typical social groups in China to facilitate explorations about stereotypes in China; this was investigated in study 2. Both studies were approved by the Academic Ethical Committee of Ningbo University, and all participants signed an informed consent form. Based on previous findings in China, we predicted that (1) the SCM could be successfully applied in mainland China; (2) most social groups receive ambivalent stereotypes; (3) in-group or social reference groups are rated as both warm and competent; (4) warmth is positively associated with competence; (5) status is positively related with competence and competition is negatively correlated with warmth.

## 2. Study 1. Stereotypes of Social Groups in Mainland China in Terms of Warmth and Competence

### 2.1. Methods

#### 2.1.1. Pretest

To identify a series of social groups which are culturally salient and unanimously shared among participants, 200 undergraduates were recruited and instructed to answer the following open-ended question adapted from Fiske et al.: “*Off the top of your head, what various types of people do you think today’s Chinese society categorizes into groups (i.e., based on age, gender, income, occupation, residences, social class, etc.)?*” [3]. After discarding incomplete questionnaires, 184 valid datasets from 184 undergraduates (84 male, 95 female, and 5 unreported gender, *M*_age_ = 21.25) were available for formal analysis. We selected groups listed by at least 10% of participants [12]. The chosen social groups and the mentioned frequency are listed in Table 2.

#### 2.1.2. Participants

We recruited 1403 undergraduates from several universities and obtained valid data from 1315 respondents (93.73%) after excluding incomplete data. To further explore the relation between social structure (status and competition) and stereotype content (warmth and competence), an additional 287 undergraduates were enrolled to evaluate the perceived status and competition for each social group, and we obtained valid data from 268 (96.38%) participants after excluding inattentive data.

#### 2.1.3. Materials and Procedures

Following Leach et al., we reviewed the literature containing warmth-related and competence-related items and selected the 18 most frequently used items [73]. For the warmth dimension, we selected *warm*, *trustworthy*, *friendly*, *good-natured*, *sincere*, *well-intentioned*, *honest*, *upright*, and *accommodating*. For the competence dimension, we selected *competent*, *confident*, *independent*, *competitive*, *intelligent*, *capable*, *skillful*, *talented*, and *conscientious*.

Next, we developed a questionnaire based on the above 18 items. Respondents were asked to evaluate 18 sentences on whether they embodied the 18 items; a 5-point Likert scale was used (ranging between 1—not at all and 5—extremely) for each group. Specifically, for each group the respondents were asked to “*Think about how [group] are viewed by people in China in general. To what extent is [group] considered by most people to be [list of items].*” (see Table 3). Cultural stereotypes rather than personal stereotypes were emphasized in the instruction. To avoid fatigue, participants were randomly assigned to answer one of nine sequences, each consisting of four or five groups. Most of the respondents were able to complete the test within 10 min.

To further explore the relationships between social structure and stereotype content, three items for perceived status and four items for perceived competition were retrieved from Fiske et al. [3] and Wu et al. [47] (see Table 3), and these items were rated for each group. Another sample of 287 undergraduate students was recruited, and 268 valid data sets were obtained. Most of the respondents were able to complete the test within 10 min.

### 2.2. Results

#### 2.2.1. Item Selection and Internal Consistency of Scales

First, principal component analysis and oblique rotation were used on all the ratings of the 18 selected items. These items could be best summarized using a two-factor solution. We deleted items with the following criteria: (1) factor loading < 0.40; (2) high factor loadings on both the warmth dimension and competence dimension. Thus, ten items were retained for further analyses, namely: *good-natured*, *well-intentioned*, *sincere*, *friendly*, and *trustworthy* (warmth dimension, α = 0.86), and *competent*, *intelligent*, *capable*, *skillful*, and *confident* (competence dimension, α = 0.84). Similarly, the seven items from the status and competition scales could be best described using a two-factor solution as predicted, that is, three items (*prestigious in the jobs*, *economically successful*, and *education level*) for status (α = 0.62) and four items (*special breaks*, *power*, *resources*, and *values and beliefs*) for competition (α = 0.85).

#### 2.2.2. Content of Stereotypes in Mainland China

**Figure 1:**
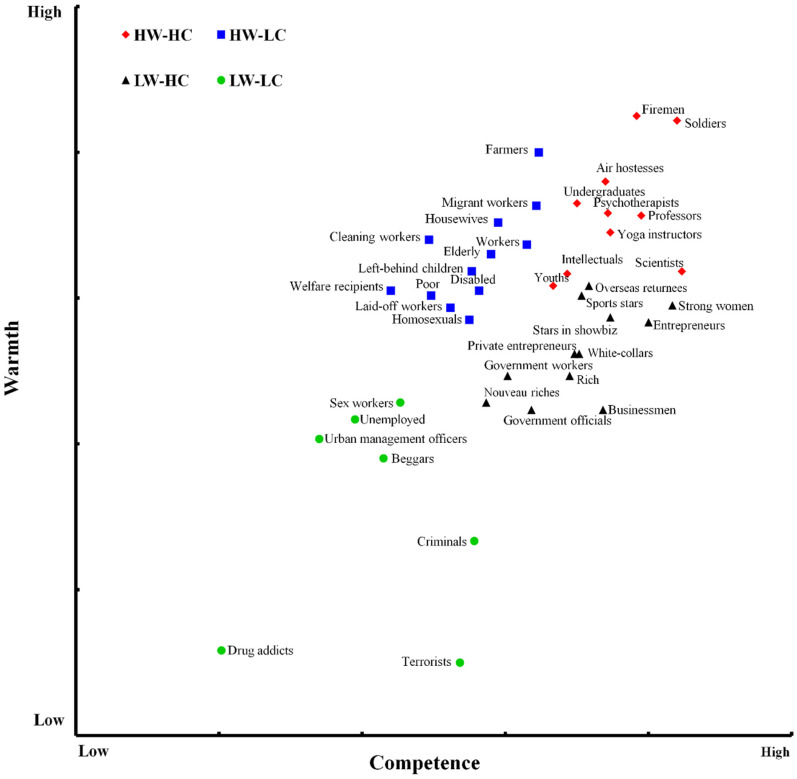
SCM map in mainland China.

To produce a stereotype map for mainland China, the warmth and competence scores for each of the 41 groups were averaged across respondents, so the means provided competence and warmth scores for each group. According to these means, the 41 groups were spread across a two-dimensional Competence × Warmth space (see Figure 1). As predicted, the two dimensions differentiated the groups.

To determine the number of clusters that best summarized the present data, a hierarchical cluster analysis (Ward method) was performed [74]. A subsequent k-means cluster analysis determined the optimal allocation of groups in the clusters. A four-cluster solution was chosen based on the agglomeration schedule. The largest cluster contained 12 groups: businessmen, overseas returnees, government workers, government officials, stars in showbiz, rich, white-collars, strong women, entrepreneurs, private entrepreneurs, sports stars, and nouveau riches. A second cluster also included 12 groups: elderly, farmers, housewives, migrant workers, left-behind children, cleaning workers, workers, disabled, poor, welfare recipients, homosexuals, and laid-off workers. A third cluster comprised soldiers, firemen, professors, psychotherapists, air hostesses, yoga instructors, undergraduates, scientists, intellectuals, and youths. The last cluster had seven groups, criminals, unemployed, beggars, drug addicts, terrorists, urban management officers, and sex workers.

Two statistical tests validated the four-cluster solution. First, a two-way 4 (clusters) × 2 (stereotype dimensions) ANOVA revealed a main effect of cluster, *F*(3, 37) = 41.88, *p* < 0.001, partial η^2^ = 0.77; a main effect of dimension, *F*(1, 37) = 13.39, *p* = 0.001, partial η^2^ = 0.27; and most importantly, an interaction of cluster by dimension, *F*(3, 37) = 17.46, *p* < 0.001, partial η^2^ = 0.59. Follow-up univariate analyses revealed significant main effects of cluster on both competence, *F*(3, 37) = 32.51, *p* < 0.001, partial η^2^ = 0.73 and warmth, *F*(3, 37) = 35.02, *p* < 0.001, partial η^2^ = 0.74, supporting both dimensions as combining to classify target groups.

#### 2.2.3. Stereotype Ambivalence

We hypothesized that most social groups in mainland China are subject to ambivalent stereotypes. We tested our hypothesis at two levels: the group level and the cluster level [75]. Groups received ambivalent stereotypes if their competence and warmth scores significantly differed. Paired-sample *t*-tests within groups revealed that 35 of the 41 target groups (85.37%) differed on the competence and warmth dimensions, *p*s < 0.01 (see Table 4).

At the cluster level, competence and warmth were compared within clusters (paired *t* test) and between clusters (one-way ANOVAs, post hoc Bonferroni’s correction). We selected the ambivalent groups according to the following criteria: only within-cluster warmth and competence comparisons, as well as between-cluster, within-dimension (high-low) comparisons, with *p* < 0.05, were considered ambivalent. That is, clusters received ambivalent stereotypes if they (1) significantly differed in competence and warmth within clusters and (2) were higher on their high dimension than groups low on that dimension and lower on their low dimension than groups high on that dimension [6,75]. To see if clusters met the first requirement, we conducted paired-sample *t*-tests within clusters, which revealed that two clusters (i.e., LW-HC and HW-LC) differed on the two dimensions, *p*s < 0.001 (see Table 5).

To see if clusters met the second requirement, we conducted one-way ANOVAs between clusters on each dimension. Post hoc multiple comparisons indicated that for the LW-HC cluster, its Competence score was significantly higher than that on LC clusters (HW-LC and LW-LC), *p*s < 0.001; its Warmth score was significantly lower than that on HW clusters (HW-HC and HW-LC), *p*s < 0.001. For the HW-LC cluster, its Warmth score was significantly higher than that on LW clusters (LW-HC and LW-LC), *p*s < 0.004; its Competence score was significantly lower than groups in HW clusters (HW-HC and LW-HC), *p*s < 0.001.

Analyses confirmed that 24 groups in mainland China met the above two criteria, that is, 12 groups in the LW-HC cluster and 12 groups in the HW-LC cluster received ambivalent stereotypes. Overall, findings revealed that the respondents did not perceive the richest groups (e.g., rich, stars in showbiz, businessmen, entrepreneurs, nouveau riches) to be equally as competent as warm but that they perceived them to have a high level of competence and low level of warmth. Inversely, the respondents did not perceive the poorest but kindly groups (e.g., elderly, farmers, housewives, migrant workers, left-behind children, cleaning workers, workers, disabled, poor, welfare recipients) to be equally as competent as they are warm but they perceived them to have a high level of warmth and low level of competence.

In addition, group-level correlation analysis revealed that perceived warmth and competence were positively correlated, *r*(41) = 0.585, *p* < 0.001. Correspondingly, as presented in Figure 1, the distribution of the groups in the W × C space presented a univalent vector pattern. As previously reported [5], the overall W-C correlation indexed ambivalence: lower W-C correlations indicated more ambivalence, whereas higher and positive W-C correlations indicated less ambivalence at a societal level. Overall, the results from the cluster-level test for ambivalent stereotypes and correlation consistently showed a low level of ambivalence at a societal level in mainland China.

#### 2.2.4. Social Structural Correlates of Warmth and Competence

**Figure 2:**
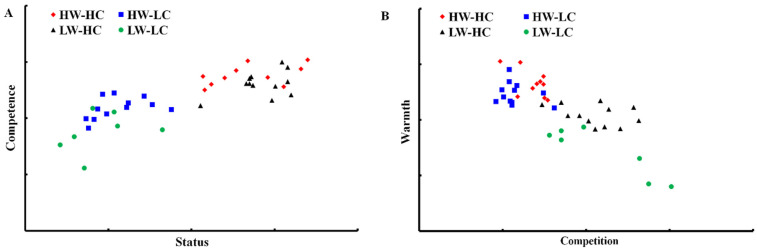
Social structural correlates of warmth and competence. (**A**): the association between competence and status; (**B**): the correlation between warmth and competition.

According to the SCM [3,76], social structure could predict stereotype dimensions. We hypothesized that perceived status and competition would correlate with perceived competence and warmth, respectively. As predicted, status and competence were positively related, *r*(41) = 0.81, *p* < 0.001; this means that the more a group has status, the more it receives competence attributions (see Figure 2A). In addition, as predicted, competition and warmth were negatively related, *r*(41) = −0.77, *p* < 0.001; as Figure 2B shows, the more a group seems competitive, the less it receives warmth attributions.

## 3. Study 2. Determination of Stereotype Words for Typical Social Groups in China

In study 1, to produce the SCM map, the items on competence and warmth were pre-determined by reviewing related literature about these two dimensions. However, some notable differences exist among social groups even though they belong to the same SCM quadrant, and the stereotypes about these typical social groups may not be limited to the ten items selected in study 1. Study 2 aimed to provide a more comprehensive picture of the stereotypes of those typical groups in China.

**Figure 3:**
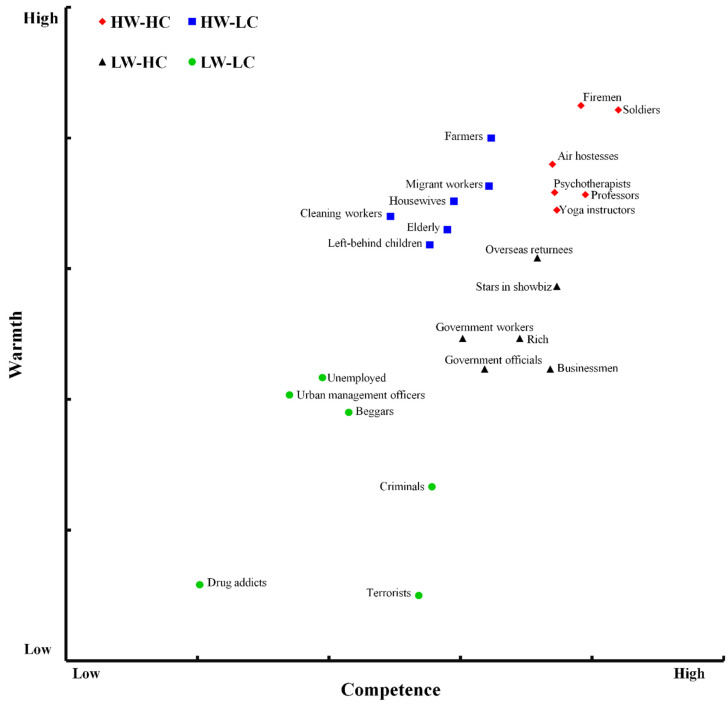
SCM map in mainland China based on 24 typical social groups.

First, we selected the six most mentioned social groups in each SCM cluster (see Table 6) and obtained a more clearly divided SCM map. Four clearly divided clusters are identified in Figure 3, with perceived competence and warmth differentiated among 24 groups.

Second, for the 24 typical groups, we adopted the classical method of Katz and Braly (1933) to determine the stereotype words for each social group [77]. The list of stereotype words would be of vital value when exploring stereotype-related processing, such as stereotype activation, stereotype application, impression management, and so on.

### 3.1. Methods

#### 3.1.1. Participants

Fifty-six undergraduates were recruited to write down stereotypes of 24 social groups. After deleting eight incomplete questionnaires, valid data from 48 respondents (85.71%, 26 female and 22 male) were retained for formal analysis.

#### 3.1.2. Materials and Procedures

Each participant was instructed to answer the following open-ended questions: “*Think about how [group] are viewed by people in China in general; please write as many words as you think are necessary to characterize these people adequately.*” This was repeated for the 24 typical social groups identified in study 1. After collecting all the stereotype words, we calculated the frequency of each word for each social group. If two words covered a similar meaning, we replaced the less frequently mentioned one with a more frequently mentioned one. We identified 210 frequently mentioned (more than three times) stereotype words in total and divided them into two subsets. Another 60 undergraduates were randomly assigned to one of the subsets. To maximize variance, they were asked to rate the typicality of stereotype words on a 7-point Likert scale, as recommended in previous studies [78,79]; the following question was used: “How typical does [stereotype word] describe [social group]?” The Likert scale ranged from 1 (“not at all”) to 7 (“very much”). We received valid data from 52 respondents (86.67%, 27 female and 25 male).

### 3.2. Results

We selected the 30 most typical stereotype words for each SCM quadrant. The descriptive data on the typicality of selected stereotype words are shown in Table 7. No significant difference was revealed on the typicality of stereotype words among the four SCM quadrants (*M*_HW-HC_ = 5.89, *SD*_HW-HC_ = 0.32; *M*_HW-LC_ = 5.99, *SD*_HW-LC_ = 0.29; *M*_LW-HC_ = 5.82, *SD*_LW-HC_ = 0.31; *M*_LW-LC_ = 5.84, *SD*_LW-LC_ = 0.43), *F* (3, 87) = 1.6, *p* = 0.196, partial η^2^ = 0.05.

## 4. Discussion

Extending the research of previous studies, we increased the sample size in both the pilot study and the formal study to 184 and 1315 respondents, respectively, and conducted the studies in 2018 to produce the most recent SCM map in mainland China. To our knowledge, this SCM validation study employed the largest sample size in mainland China to date. The findings support the cross-cultural generalizability of the SCM [3,4,5,6,7,16,17] and validate previous research in China [6,47,49,50,51,52]. As predicted, 35 of 41 groups received ambivalent stereotype, and in-group (undergraduates) and social reference groups were assessed as both high in the warmth dimension and high in the competence dimension.

### 4.1. Classical Social Groups in SCM Map in China

Twenty-nine out of 41 social groups (70.73%) in the current study overlap at least once with social groups in previous studies (see Table 8). For some frequently appeared social groups, the findings replicated those of previous studies; for example, elderly people, welfare recipients, laid-off workers, and homosexuals were categorized into the HW-LC quadrant, while unemployed people, beggars, and criminals were clustered into the LW-LC quadrant. Professors were categorized into the HW-HC quadrant. Sports stars, entrepreneurs, and overseas returnees were categorized into the LW-HC quadrant. Note that Shi and Wang [52] labeled SCM quadrants according to the relative location of each group on the SCM map; in their study, all social groups actually ended up in the HW-HC quadrant based on their warmth and competence rating scores. Despite the similarities, some variations appeared. For instance, rich, white-collars, and businessmen were grouped into the MW-HC quadrant in Wu et al. [47], while these three groups belonged to the LW-HC quadrant in the current study, which replicates findings from most previous studies [6,49,50,51]. When examined in detail, the rating scores for the MW-HC quadrant in Wu et al. (competence = 3.55, warmth = 2.96) were very similar to the scores for the LW-HC quadrant in the current study (competence = 3.57, warmth = 3.03). Thus, our results for rich people are similar to rather than different from Wu et al. Government officials and government workers were both rated as low in warmth, while the ratings on competence were not consistent [47,49,50,51]. This suggests that these two groups should improve their attitude when providing services and that the Chinese government needs to pay more attention to warmth information when constructing a public image. Regarding poor people, the results were more inconsistent: our findings support Guan and Cheng, where this group is a HW-LC group, while it is a LW-LC group in Cuddy et al. [6] and a MW-LC group in Wu et al. [47]. Some minor variations also exist for the remaining groups; one reason for this might be the differences in the listed groups among studies, as stated in the introduction. Another reason might be the existence of subgroups of these groups, which was demonstrated by Wu et al. [47].

### 4.2. Emerging Social Groups on the SCM Map in China

More importantly, 12 new groups appeared on the SCM map in our findings, such as air hostesses, firemen, yoga instructors, and psychotherapists in the HW-HC quadrant; cleaning workers and left-behind children in the HW-LC quadrant; nouveau riches and strong women in the LW-HC quadrant; and sex workers, urban management workers, drug addicts and terrorists in the LW-LC quadrant. These emerging social groups may reflect some important recent changes in China. For example, the increasing prevalence of mental disorders in China during recent years [80] has made people more inclined to seek help from psychotherapists. The development of the economy has made psychological services affordable, meaning psychotherapists can be more easily and frequently accessed than even before. Regarding urban management workers, the frequently reported violence between urban management workers and street vendors [81] on social media makes this group vulnerable to substantial stigma, and thus leads to it falling into the LW-LC quadrant. With the urbanization of China over the past decades, millions of migrant workers have flocked to the cities and this group has attracted much attention in previous studies [47,49,50]. However, their children who were left behind in rural areas were neglected in former studies. As they grew older, they became second-generation migrant workers or went to colleges to receive higher education. The increasing number of left-behind children and the poor psychological and behavioral outcomes found in this group [82] have made this group increasingly visible during recent years. The left-behind children were classified as a HW-LC group, as were children in past studies [6,51]; however, it should be noted that this group is quite different from normal children. They are lonely, pitiful, experience a lack of love and care, and are eager to be accompanied by parents as the results from study 2 indicate. Thus, this group deserves more attention in future studies. Drug addicts and terrorists belong to the LW-LC quadrant, which replicates the findings of previous studies in other countries [10,15]. The reasons why drug addicts and terrorists appear on SCM map might be due to the increasing use of drugs [83] and mounting media coverage about illicit drugs [84] and terrorist attacks [85] in China.

We calculated the interrater agreement (Kendall’s W) on warmth, competence, status, and competition for each social group (see Table S1 in the Supplementary Material). Overall, a satisfactory interrater agreement was achieved on four dimensions for each social group (*p*s < 0.01); only stars in showbiz and nouveau riches received low interrater agreement on status (*p*s > 0.05). The results show that the highest agreement appeared for terrorists and the lowest agreement appeared for overseas returnees on warmth; the highest agreement appeared for strong women and the lowest agreement appeared for elderly on competence; the highest agreement appeared for clearing workers and the lowest agreement appeared for stars in showbiz on status; the highest agreement appeared for terrorists and the lowest agreement appeared for professors on competition. The SCM method asks for society’s views, so it tends to emphasize a culturally shared lay theory of groups in society. Indeed, as suggested elsewhere, people from the same culture, whether actively biased or not, know their own cultural stereotypes [86,87]. However, interrater difference still exists and needs special attention when exploring stereotype content. Although stars in showbiz and nouveau riches obtained satisfactory interrater agreement on warmth and competence, the low interrater agreement they obtained on status may indicate there are many subgroups within each social group. Previous subgroup (such as gender [88], gay men [89], immigrants [75,90], refugees [91,92], native Americans [93], and the rich [47]) analysis supports this possibility. Further studies should be conducted on these two groups and other social groups with low interrater agreement.

Contrary to previous studies [6,47], warmth and competence was positively related (r = 0.585) in our study, which was similar to Denmark (r = 0.58) [5]. Since peace-conflict predict predicted stereotype ambivalence and both extremely peace nations and extremely conflict nations showed unambivalence [5]. This increase in unambivalence might indicate that whether the Chinese society has become more peaceful or more conflict during recent years. Which one is true needs further investigation. We found that status was positively related to competence and competition is negatively associated with warmth, which supported our hypothesis and confirmed SCM predictions [3,6].

Alongside the validation study of the SCM in mainland China, we developed a stereotype word list for 24 typical social groups in study 2, which was successfully used in Yang et al. to explore the behavioral patterns of stereotype activation among four SCM quadrants [94] and in their follow-up study using event-related potential technology [95]. This stereotype word list could be applied in multiple mental processes involved in stereotypes, such as stereotype activation [96,97], stereotype application, and impression formation.

Some limitations should be mentioned and addressed in future research. First, we only recruited undergraduates in the current investigation; a representative community sample with children or adolescents would provide more insights into stereotype content in China. Second, the present research only examined the SCM map in mainland China, the emotion and behavioral tendencies related to various social groups were not explored, so the Behaviors from Intergroup Affect and Stereotype (BIAS) map [21] based on the SCM could not be tested in the current study and needs to be examined in the future. With the development of technology, analysis of big data from multiple sources has become feasible and easy to achieve. Previous validation studies of the SCM mainly employed a theory-driven method to test the hypotheses retrieved from the SCM; more data-driven methods should be utilized to analyze the stereotype content in future studies. The recently developed R package Semi-Automated Dictionary Creation for Analyzing Text [98] is an excellent example to explore and develop stereotype content dictionaries. New data analysis methods (such as network analysis [14,99,100]) would provide novel insights into stereotype content from the traditional data obtained from questionnaires. Implicit stereotype content was found to be different from explicit stereotype content [35,101,102,103]. Considering that explicit stereotypes have been extensively explored in China, implicit stereotype content warrants further investigation. Previous studies found that the research method would impact stereotype content [104]; thus, a mixed-method paradigm [46,105] is encouraged for use in further explorations to achieve a more comprehensive picture of stereotype content.

## Figures and Tables

**Table 1:** Summary of validation studies on Stereotype Content Model in China.

Reference	Region	Sample	Sample Size of Pretest	Sample Size of Formal Test	Number of Groups	SCM Quadrants: Social Groups Included
[6]	Hong Kong	Undergraduates	30	60	27	HW-LC: children, elderly, Christians, and mentally ill; LW-HC: professionals, and rich; LW-LC: janitors, poor, Filipino maids, unemployed, immigrants, mainlanders, and Pakistani; MW-MC: foreigners, students, women, Asians, married, Chinese, college grads, youths, adults, HK locals, blue-collars, white-collars, singles, and men.
[51]	Mainland China	Undergraduates	23	385	18	HW-HC: blue-collars, farmers, undergraduates, disabled, and housewives; HW-LC: children, elderly, and welfare recipients; LW-HC: businessmen, white-collars, rich, men, intellectuals, and professional women; LW-LC: beggars, unemployed, homeless, and government officials/politicians.
[49]	Mainland China	Undergraduates	115	160	21	HW-HC: doctors, self-employed, intellectuals, college teachers, technical workers, and white-collars; HW-LC: farmers, elderly, disabled, migrant workers, homosexuals, religious believers, and unemployed; LW-HC: rich, government officials/politicians, and stars in showbiz; MW-MC: undergraduates, national minorities, policemen, soldiers in People’s Liberation Army, and teenagers born in the 80s.
[50]	Mainland China	Undergraduates	125	103	32	HW-HC: teachers, blue-collars, women, undergraduates, northerners, resident foreigners, and self-employed; HW-LC: elderly, farmers, poor, migrant workers, welfare recipients, disabled, and unemployed; LW-HC: rich, men, businessmen, overseas returnees, white-collars, government workers, urban residents, scientists, entrepreneurs, southerners, leaders, intellectuals, stars in showbiz, sports stars, and private business owners; LW-LC: criminals, beggars, and unemployed.
[52]	Mainland China	Community sample	—	4659	11	HW-HC: scientists, and engineers; HW-LC: military officers, journalists, doctors, and private entrepreneurs; LW-HC: secondary school teachers, technical workers, and agricultural technology extension workers; LW-LC: government workers, and stars.
[47]	Mainland China	Online sample	25	199	22	HW-HC: blue-collars, women, workers, migrant workers, and intellectuals; LW-MC: government officials/politicians, and government workers; MW-HC: rich, businessmen, doctors, white-collars, men, adults, employees, and urban residents; HW-MC: children, students, adolescents, and rural residents; MW-LC: poor, and disabled; LW-LC: unemployed.

**Table 2:** Frequency of selected social groups (%).

Group Names in English (in Chinese)	%	Group Names in English (in Chinese)	%	Group Names in English (in Chinese)	%
Government workers (公务员)	72.80	Poor (穷人)	50.91	Air hostesses (空姐)	28.86
Migrant workers (农民工)	70.48	Entrepreneurs (企业家)	49.28	Disabled (残疾人)	26.70
Undergraduates (大学生)	70.19	Housewives (家庭主妇)	48.39	Urban management officers (城管)	25.17
Stars in showbiz (演艺明星)	68.51	Professors (大学教师)	46.96	Criminals (罪犯)	24.89
White-collars (白领)	66.88	Soldiers (军人)	43.19	Laid-off workers (下岗工人)	23.32
Elderly (老年人)	65.84	Psychotherapists (心理咨询师)	41.25	Nouveau riches (暴发户)	20.71
Government officials (政府官员)	64.11	Intellectuals (知识分子)	40.41	Terrorists (恐怖分子)	18.94
Rich (富人)	62.71	Yoga instructors (瑜伽教练)	39.24	Homosexuals (同性恋)	17.19
Scientists (科学家)	60.49	Beggars (乞丐)	38.10	Drug addicts (吸毒者)	16.45
Businessmen (商人)	59.88	Workers (工人)	36.72	Welfare recipients (低保人员)	15.05
Farmers (农民)	58.87	Sports stars (体育明星)	35.14	Private entrepreneurs (私营企业主)	14.15
Youths (年青人)	56.22	Unemployed (无业游民)	33.21	Strong women (女强人)	12.58
Cleaning workers (清洁工)	54.31	Overseas returnees (海归)	30.29	Sex workers (性工作者)	10.95
Left-behind children (留守儿童)	53.93	Firemen (消防员)	29.04		

**Table 3:** Scale items for competence, warmth, status and competition.

Construct	Items
Competence	Think about how [group] are viewed by people in China in general. To what extent is [group] considered by most people to be [*warm, trustworthy, friendly, good-natured, sincere, well-intentioned, honest, upright, accommodating*]?
Warmth	Think about how [group] are viewed by people in China in general. To what extent is [group] considered by most people to be [*competent, confident, independent, competitive, intelligent, capable, skillful, talented, conscientious*]?
Status	How prestigious are the jobs typically achieved by members of [group]?
	How economically successful have members of [group] been?
	How well educated are members of this [group]?
Competition	If members of [group] get special breaks (such as preference in hiring decisions), this is likely to make things more difficult for people like me.
	The more power members of [group] have, the less power people like me are likely to have.
	Resources that go to members of [group] are likely to take away from the resources of people like me.
	The values and beliefs of [group] are not compatible with the beliefs and values of most Chinese.

**Table 4:** Paired competence-warmth differences, by group.

Groups	Competence *M* (*SD*)	Warmth *M* (*SD*)	*df*	Difference	*t*	Cohen’s *d*
Terrorists	3.11 (0.92)	1.80 (0.85)	146	1.31	14.07 ***	1.16
Businessmen	3.71 (0.66)	2.84 (0.65)	146	0.87	11.80 ***	0.97
Criminals	3.17 (0.89)	2.30 (0.83)	165	0.87	11.14 ***	0.86
Strong women	4.00 (0.66)	3.27 (0.62)	146	0.73	12.35 ***	1.02
Entrepreneurs	3.90 (0.74)	3.19 (0.72)	133	0.71	9.60 ***	0.83
Scientists	4.04 (0.67)	3.41 (0.64)	169	0.63	10.97 ***	0.84
Rich	3.57 (0.71)	2.98 (0.62)	169	0.59	9.35 ***	0.72
Government officials	3.41 (0.74)	2.84 (0.76)	175	0.57	10.27 ***	0.77
White-collars	3.61 (0.63)	3.07 (0.60)	146	0.54	10.35 ***	0.85
Stars in showbiz	3.74 (0.74)	3.22 (0.69)	165	0.52	9.19 ***	0.71
Private entrepreneurs	3.59 (0.62)	3.07 (0.60)	146	0.52	8.70 ***	0.72
Nouveau riches	3.22 (0.66)	2.87 (0.70)	114	0.35	5.52 ***	0.51
Government workers	3.31 (0.70)	2.98 (0.71)	175	0.33	7.35 ***	0.55
Sports stars	3.62 (0.61)	3.31 (0.63)	114	0.31	5.49 ***	0.51
Overseas returnees	3.65 (0.56)	3.35 (0.50)	149	0.3	7.43 ***	0.61
Drug addicts	2.11 (0.85)	1.85 (0.80)	109	0.26	5.00 ***	0.48
Professors	3.87 (0.67)	3.64 (0.68)	146	0.23	5.21 ***	0.43
Yoga instructors	3.74 (0.62)	3.57 (0.61)	109	0.17	3.94 ***	0.38
Intellectuals	3.56 (0.62)	3.40 (0.71)	146	0.16	3.70 ***	0.30
Urban management officers	2.79 (0.72)	2.64 (0.75)	175	0.15	3.95 ***	0.30
Youths	3.50 (0.66)	3.35 (0.68)	114	0.15	2.98 **	0.28
Psychotherapists	3.73 (0.61)	3.65 (0.60)	149	0.08	2.12	0.17
Sex workers	2.86 (0.85)	2.87 (0.85)	133	−0.01	−0.27	0.02
Soldiers	4.02 (0.60)	4.03 (0.62)	175	−0.01	−0.48	0.04
Air hostesses	3.72 (0.76)	3.78 (0.68)	165	−0.06	−1.6	0.12
Homosexuals	3.15 (0.81)	3.21 (0.83)	146	−0.06	−1.79	0.15
Undergraduates	3.60 (0.65)	3.69 (0.60)	133	−0.09	−2.18	0.19
Unemployed	2.67 (0.73)	2.80 (0.71)	149	−0.13	−2.97**	0.24
Workers	3.39 (0.60)	3.52 (0.60)	133	−0.13	−3.81 ***	0.33
Disabled	3.19 (0.63)	3.33 (0.67)	165	−0.14	−3.18 **	0.25
Laid−off workers	3.07 (0.57)	3.26 (0.60)	114	−0.19	−3.71 ***	0.35
Beggars	2.52 (0.78)	2.72 (0.72)	169	−0.20	−4.13 ***	0.32
Firemen	3.85 (0.63)	4.05 (0.64)	175	−0.20	−5.94 ***	0.45
Elderly	3.24 (0.63)	3.48 (0.64)	149	−0.24	−3.82 ***	0.31
Left−behind children	3.16 (0.73)	3.41 (0.63)	133	−0.25	−5.46 ***	0.47
Migrant workers	3.43 (0.70)	3.68 (0.74)	165	−0.25	−5.85 ***	0.45
Poor	2.99 (0.64)	3.32 (0.66)	146	−0.33	−5.91 ***	0.49
Housewives	3.27 (0.66)	3.61 (0.56)	169	−0.34	−6.60 ***	0.51
Farmers	3.44 (0.75)	3.90 (0.70)	109	−0.46	−6.31 ***	0.60
Welfare recipients	2.82 (0.65)	3.33 (0.72)	146	−0.51	−8.47 ***	0.70
Cleaning workers	2.98 (0.84)	3.54 (0.73)	109	−0.56	−6.26 ***	0.60

Note: Matched pair *t*-tests revealed that competence and warmth ratings differed for 35 out of the 41 target groups. Positive differences refer to greater competence and negative to greater warmth. *** *p* < 0.001, ** *p* < 0.01.

**Table 5:** The descriptive data and paired t-test results of warmth and competence for four social group clusters (based on 41 social groups).

Social Groups	Label of Cluster	Competence *M* (*SD*)		Warmth *M* (*SD*)	*t*	*p*	Cohen’s *d*
soldiers, firemen, professors, psychotherapists, air hostesses, yoga instructors, undergraduates, scientists, intellectuals, youths	HW-HC	3.76 (0.18)	=	3.66 (0.24)	1.45	0.182	0.46
businessmen, overseas returnees, government workers, government officials, stars in showbiz, rich, white-collars, strong women, entrepreneurs, private entrepreneurs, sports stars, nouveau riches	LW-HC	3.61 (0.22)	>	3.08 (0.18)	10.02	< 0.001	2.89
elderly, farmers, housewives, migrant workers, left-behind children, cleaning workers, workers, disabled, poor, welfare recipients, homosexuals, laid-off workers	HW-LC	3.18 (0.19)	<	3.47 (0.20)	−6.40	< 0.001	1.85
criminals, unemployed, beggars, drug addicts, terrorists, urban management officers, sex workers	LW-LC	2.75 (0.36)	=	2.43 (0.44)	1.52	0.179	0.58

**Table 6:** The descriptive data and paired *t*-test results of warmth and competence for four social group clusters (based on 24 groups).

Social Groups	Label of Cluster	Competence *M* (*SD*)		Warmth *M* (*SD*)	*t*	*p*	Cohen’s *d*
soldiers, firemen, professors, psychotherapists, air hostesses, yoga instructors	HW-HC	3.82 (0.12)	=	3.79 (0.21)	0.51	0.632	0.21
businessmen, overseas returnees, government workers, government officials, stars in showbiz, rich	LW-HC	3.57 (0.17)	<	3.03 (0.21)	−6.31	0.001	2.58
elderly, farmers, housewives, migrant workers, left-behind children, cleaning workers	HW-LC	3.26 (0.20)	>	3.61 (0.18)	−6.58	0.001	2.70
criminals, unemployed, beggars, drug addicts, terrorists, urban management officers	LW-LC	2.73 (0.39)	=	2.35 (0.44)	−1.56	0.180	0.64

**Table 7:** The descriptive data of ratings on typicality of stereotype words for 24 typical social groups.

SCM Quadrant	Group Names in English (in Chinese)	Stereotype Words in English (in Chinese)	*M* (*SD*)
HW-HC	Soldiers (军人)	Protect our homes and defend our country (保家卫国)	5.18(1.43)
		Dignified (威严)	5.44(1.60)
		Fortitude (坚毅)	5.42(1.67)
		Righteous (正义)	5.33(1.67)
		Upstanding (正直)	5.25(1.71)
	Firemen (消防员)	Dangerous work (工作危险)	6.33(0.90)
		Act quickly (行动迅速)	6.21(0.91)
		Put out the fire and rescue people (灭火救人)	6.15(0.98)
		Respectable (令人尊敬)	6.04(1.05)
		Dedicated (敬业)	5.96(1.05)
		Brave (勇敢)	5.96(0.99)
	Professors (大学教师)	Scholarly (有学问)	6.01(1.15)
		Knowledgeable (知识渊博)	5.81(1.23)
		Imparting knowledge and educating people (教书育人)	5.74(1.14)
	Psychotherapists (心理咨询师)	Good at observing (善于观察)	5.96(0.91)
		Empathetic (懂人心)	5.65(1.10)
		Have much patience (有耐心)	5.52(0.96)
		Gentle (温和)	5.46(1.07)
		Helping others (帮助他人)	5.31(1.23)
	Air hostesses (空姐)	Good manners (礼仪好)	6.15(1.02)
		Service with a smile (微笑服务)	6.13(0.97)
		Graceful (有气质)	6.10(1.07)
		Pretty (漂亮)	6.06(0.89)
		Beautiful and gracious (美丽大方)	5.98(1.15)
		Elegant manner (举止优雅)	5.98(1.08)
	Yoga instructors (瑜伽教练)	Great flexibility (柔韧性强)	6.33(0.88)
		Beautiful shape (体形优美)	6.23(0.90)
		Good figure (身材好)	6.12(0.94)
		Well-built (健美)	6.06(0.92)
		Athletic (健康)	6.06(1.04)
HW-LC	Elderly (老年人)	Need to be looked after (需要照顾)	6.17(0.90)
		Need love and care (需要关爱)	6.10(0.87)
		Nagging (爱唠叨)	5.87(1.05)
		Grey-haired (白发苍苍)	5.83(0.94)
		Slow movement (行动缓慢)	5.73(1.24)
		Kindly (慈祥)	5.37(1.44)
	Farmers (农民)	Laborious work (辛苦)	6.25(0.95)
		Farming (种田)	6.12(1.04)
		Industrious (勤劳)	6.04(1.20)
		Simple and honest (憨厚老实)	5.96(1.19)
		Low-income (收入低)	5.79(1.29)
	Housewives (家庭主妇)	Cooking and taking care of children (做饭带孩子)	5.88(1.28)
		Value family (顾家)	5.87(1.17)
		Keep house (做家务)	5.84(1.18)
		Virtuous (贤惠)	5.71(1.25)
		Industrious (勤劳)	5.53(1.18)
	Migrant workers (农民工)	Laborious work (辛苦)	6.48(1.02)
		Overworked (劳累)	6.27(1.05)
		Life is tough (生活艰辛)	6.13(1.12)
		Vulnerable group (弱势群体)	6.08(1.15)
	Left-behind children (留守儿童)	Eager to be accompanied by parents (渴望陪伴)	6.46(1.00)
		Lack of love and care (缺少关爱)	6.13(1.28)
		Lonely (孤独)	6.12(1.13)
		Pitiful (可怜)	5.98(1.23)
	Cleaning workers (清洁工)	Laborious work (辛苦)	6.47(0.94)
		Early rising (早起)	6.41(0.97)
		Simple (朴实)	5.41(1.32)
		Worthy of respect (可敬)	5.92(1.30)
		Industrious (勤劳)	5.80(1.25)
		Low salary (工资低)	5.57(1.46)
LW-HC	Businessmen (商人)	Regard interests as highly important (重利益)	5.81(1.43)
		Wealthy (有钱)	5.44(1.60)
		Shrewd (精明)	5.42(1.67)
		Seek nothing but profits (唯利是图)	5.33(1.67)
	Overseas returnees (海归)	Study abroad (留学)	5.57(1.54)
		Good foreign language (外语好)	5.51(1.41)
		Well-informed (见识广)	4.80(1.14)
		Educated (有知识)	4.69(1.21)
	Government workers (公务员)	Stable job (工作稳定)	6.21(1.04)
		Has a stable lifelong job (铁饭碗)	6.08(1.27)
		Work at leisure (工作清闲)	5.90(1.30)
		Well-paid (待遇好)	5.85(1.35)
	Government officials (政府官员)	Official jargon (官腔)	6.06(1.07)
		Formalism (形式主义)	6.02(1.24)
		Bureaucratic (官腔官调)	6.00(1.17)
		Shout slogans (喊口号)	5.96(1.19)
		Don’t get things done (不办实事)	5.62(1.36)
		Corrupt (贪污腐败)	5.48(1.32)
		Big-bellied (大腹便便)	5.75(1.08)
	Stars in showbiz (演艺明星)	High income (收入高)	6.33(0.86)
		More scandal (绯闻多)	5.92(1.10)
		Glamorous (光鲜亮丽)	5.71(1.19)
		Pretty (漂亮)	5.71(1.18)
		Promiscuous (私生活混乱)	5.46(1.45)
		Good-looking (好看)	5.56(1.24)
	Rich (富人)	Wealthy (有钱)	6.25(1.03)
		Extravagant (奢侈)	5.40(1.42)
		Arrogant (傲慢)	5.02(1.34)
		Crafty (狡猾)	4.90(1.11)
		Spend extravagantly (挥霍)	4.87(1.25)
LW-LC	Criminals (罪犯)	Prison (监狱)	5.71(1.50)
		Hateful (可恶)	5.25(1.52)
		Fierce (凶狠)	5.00(1.53)
		Violent and cruel (残暴)	4.94(1.50)
	Unemployed (无业游民)	Idle (游手好闲)	6.04(1.20)
		Slothful (无所事事)	5.85(1.29)
		Irresponsible (无责任心)	5.56(1.43)
		Depend on parents for living (啃老)	5.54(1.47)
		No desire to advance (没有上进心)	5.50(1.53)
	Beggars (乞丐)	Dirty (脏)	5.49(1.39)
		Shabbily dressed (衣衫褴褛)	5.47(1.53)
		Slovenly (邋遢)	5.27(1.36)
		Lazy (懒惰)	4.76(1.48)
		Poor (贫穷)	4.76(1.42)
	Drug addicts (吸毒者)	Decadence (颓废)	6.37(0.91)
		Decadent (堕落)	6.25(1.28)
		Flattened (萎靡)	6.23(1.13)
		Spiritless (精神涣散)	6.21(1.19)
		Skinny (骨瘦如柴)	6.21(1.16)
		Haggard (形容枯槁)	6.19(1.30)
		Thin and weak (瘦弱)	6.12(1.25)
	Terrorists (恐怖分子)	Go to extremes (极端)	6.37(1.05)
		Brutal (残暴)	6.29(1.07)
		Cruel (凶残)	6.12(1.25)
		Psychological distortion (心理扭曲)	5.88(1.38)
		Inhuman (没人性)	5.85(1.41)
	Urban management officers (城管)	Bully the weak and fear the strong (欺软怕硬)	5.40(1.59)
		Rude and unreasonable (蛮横)	5.27(1.60)
		Merciless (凶残)	5.13(1.53)
		Without compassion (没有同情心)	5.13(1.51)

**Table 8:** Classical social groups and their SCM quadrants in China.

	Cuddy (2005) [6]	Gao (2008) [49]	Yuan (2009) [51]	Guan (2009) [50]	Shi (2011) [52]	Wu (2014) [47]	Current Study (2018)
Rich	LW-HC	LW-HC	LW-HC	LW-HC		MW-HC	LW-HC
White-collars	MW-MC	HW-HC	LW-HC	LW-HC		MW-HC	LW-HC
Elderly	HW-LC	HW-LC	HW-LC	HW-LC			HW-LC
Unemployed	LW-LC		LW-LC	LW-LC		LW-LC	LW-LC
Disabled		HW-LC	HW-HC	HW-LC		MW-LC	HW-LC
Intellectuals		HW-HC	LW-HC	LW-HC		HW-HC	HW-HC
Poor	LW-LC			HW-LC		MW-LC	HW-LC
Farmers		HW-LC	HW-HC	HW-LC			HW-LC
Undergraduates		HW-MC	HW-HC	HW-HC			HW-HC
Government officials		LW-HC	LW-LC			LW-MC	LW-HC
Sports stars		LW-HC		LW-HC	LW-LC		LW-HC
Private entrepreneurs		HW-HC		LW-HC	HW-LC		LW-HC
Migrant workers		HW-LC		HW-LC		HW-HC	HW-LC
Workers		HW-HC			LW-HC	HW-HC	HW-LC
Businessmen			LW-HC	LW-HC		MW-HC	LW-HC
Government workers				LW-HC	LW-LC	LW-MC	LW-HC
Laid-off workers		HW-LC		HW-LC			HW-LC
Stars in showbiz		LW-HC			LW-LC		LW-HC
Welfare recipients			HW-LC	HW-LC			HW-LC
Beggars			LW-LC	LW-LC			LW-LC
Scientists				LW-HC	HW-HC		HW-HC
Soldiers		HW-MC					HW-HC
Professors		HW-HC					HW-HC
Homosexuals		HW-LC					HW-LC
Housewives			HW-HC				HW-LC
Criminals				LW-LC			LW-LC
Entrepreneurs				LW-HC			LW-HC
Overseas returnees				LW-HC			LW-HC
Youths						MW-MC	HW-HC

Note: The numbers in brackets indicate the year in which the data was collected. For each reference, only the first author was shown.

## Data Availability

The data used to support the findings of this study are available from the corresponding authors upon reasonable request.

## Supplementary Materials

The following are available online at https://www.mdpi.com/article/10.3390/ijerph18073559/s1, Table S1: interrater agreement (Kendall’s W) of each scale for 41 social groups.
