# Supplementary material for: Stereotypes of Social Groups in Mainland China in Terms of Warmth and Competence: Evidence from a Large Undergraduate Sample
Source: Int J Environ Res Public Health. 2021 Mar 30;18(7):3559. doi: 10.3390/ijerph18073559 (PMC8037077; doi:10.3390/ijerph18073559)
Supplement: Supplementary file 1 [file ijerph-18-03559-s001.pdf]

Table S1 Interrater agreement (Kendall's W) of each scale for 41 social groups

SCM quadrant	Social groups	Warmth	Competence	Status	Competition
	soldiers	0.682***	0.639***	0.459***	0.708***
	firemen	0.714***	0.620***	0.575***	0.800***
	professors	0.722***	0.676***	0.547***	0.603***
	psychotherapists	0.510***	0.525***	0.612***	0.646***
	air hostesses	0.625***	0.693***	0.594***	0.699***
**HW-HC**	yoga instructors	0.640***	0.641***	0.623***	0.709***
	undergraduates	0.611***	0.643***	0.517***	0.623***
	scientists	0.546***	0.618***	0.495***	0.699***
	intellectuals	0.619***	0.567***	0.596***	0.706***
	youths	0.632***	0.615***	0.553***	0.614***
	businessmen	0.571***	0.651***	0.467***	0.676***
	Overseas returnees	0.471***	0.507***	0.653***	0.678***
	government workers	0.642***	0.618***	0.645***	0.604***
	government officials	0.743***	0.656***	0.575***	0.657***
	stars in showbiz	0.590***	0.653***	0.329	0.674***
	rich	0.570***	0.644***	0.431**	0.653***
**LW-HC**	white-collars	0.621***	0.578***	0.713***	0.631***
	strong women	0.578***	0.763***	0.669***	0.722***
	entrepreneurs	0.621***	0.717***	0.525***	0.680***
	private entrepreneurs	0.653***	0.614***	0.575***	0.712***
	sports stars	0.603***	0.532***	0.464***	0.694***
	nouveau riches	0.643***	0.516***	0.362	0.698***
	elderly	0.585***	0.463***	0.582***	0.629***
	farmers	0.701***	0.566***	0.721***	0.677***
	housewives	0.576***	0.552***	0.547***	0.681***
	migrant workers	0.614***	0.531***	0.743***	0.716***
	left-behind children	0.654***	0.648***	0.713***	0.684***
	cleaning workers	0.625***	0.690***	0.799***	0.709***
**HW-LC**	workers	0.662***	0.541***	0.695***	0.749***
	disabled	0.728***	0.550***	0.639***	0.736***
	poor	0.706***	0.601***	0.610***	0.700***
	welfare recipients	0.663***	0.602***	0.767***	0.741***
	homosexuals	0.760***	0.711***	0.506***	0.693***
	laid-off workers	0.555***	0.496***	0.678***	0.739***
	criminals	0.688***	0.674***	0.563***	0.770***
	unemployed	0.624***	0.630***	0.664***	0.723***
	beggars	0.654***	0.656***	0.702***	0.718***
**LW-LC**	drug addicts	0.728***	0.693***	0.609***	0.760***
	terrorists	0.776***	0.651***	0.519***	0.805***
	urban management officers	0.755***	0.713***	0.700***	0.698***
	sex workers	0.681***	0.643***	0.505***	0.709***

Note: ***p < 0.001, **p < 0 .01, *p < 0.05
